# Bleomycin induced urethral stricture in Hodgkin's disease

**DOI:** 10.4103/0970-1591.56180

**Published:** 2009

**Authors:** Ritesh Tapkire, N. Kathiresan, B. Satheesan

**Affiliations:** Department of Surgical Oncology, Cancer Institute (W.I.A.), Adyar, Chennai, India

**Keywords:** Bleomycin, Hodgkins disease, pulmonary fibrosis, urethral stricture

## Abstract

Bleomycin is a glycoprotein that is extensively used in combination with other anti-cancer agents because of its relative lack of hematological and gastrointestinal toxicity. However, pulmonary toxicity is common with bleomycin and limits its therapeutic utility. Urethral stricture as a result of bleomycin toxicity has not been reported in literature. In this case report, a young male patient who developed urethral stricture after bleomycin-based chemotherapy is described and the possible effects of bleomycin on the urethra are discussed.

## INTRODUCTION

Bleomycin is an anti-neoplastic antibiotic complex produced by fermentation from Streptomyces verticillus.[1] Bleomycin is mainly used as part of combination regimens for the treatment of Hodgkin's and non-Hodgkin's lymphoma, germ cell tumors, squamous cell cancers of the head and neck, and squamous cell carcinomas of the skin, cervix, vulva, and penis. As a single agent, it is used as a sclerosing agent to control malignant pleural effusions and ascitis. Bleomycin is known to induce fibrosis at the region of tumor-bearing tissue and distant sites like the lungs.[2] The dose limiting toxicity of bleomycin is pulmonary toxicity. Idiosyncratic hypersensitivity reactions in the form of fever, chills, urticaria, periorbital edema, and wheezing are observed in up to 25% of the patients. We are reporting a case of urethral fibrosis probably due to bleomycin toxicity leading to stricture formation.

## CASE REPORT

**Figure 1:**
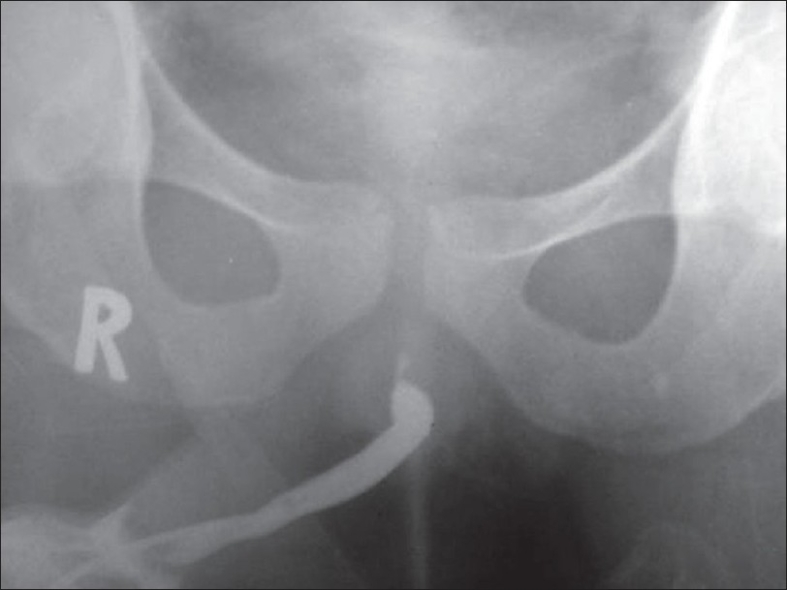
Ascending urethrogram showing the urethral stricture

A 21-year-old male presented with complaints of swelling in the neck and fever of 7 months duration. On evaluation, he was diagnosed with Hodgkin's Disease III B. He was treated with seven cycles of combination chemotherapy (total dose for all 7 cycles = Adriamycin 350 mg/m^2^, bleomycin 140 mg/m^2^, vinblastine 84 mg/m^2^, and dacarbazine 5250 mg/m^2^ ABVD) from February 2007 to August 2007. After the second cycle of chemotherapy, he developed dysuria. A burning sensation in the urethra was persistent for some time after micturition. There was no hematuria or turbid urine. His urine was sterile and the symptoms subsided after symptomatic treatment. He did not have any evidence of urological disease before the start of treatment. He had similar episodes though milder after subsequent cycles. In August 2007, after seven cycles, he presented with dyspnea and cough. A computed tomography (CT) scan of the chest showed features suggestive of bleomycin-induced pulmonary toxicity. Pulmonary function tests revealed obstructive airway disease. He was treated with antibiotics and corticosteroids. A positron emission computerised tomography (PET - CT) after seven cycles of chemotherapy was negative for any residual disease. In March 2008, he presented with burning micturition and a poor stream of urine. The stream was narrow and he had to strain. A urine culture was sterile. An ascending urethrogram showed a stricture of 1–1.5 cm in length at the level of membranous urethra extending to prostatic urethra [Figure 1]. A cystoscopy performed with a No. 17 Fr rigid cystoscope (Karl Storz, Germany) revealed a normal anterior urethra and a stricture starting in the membranous urethra. The cystoscope could not be passed beyond. An ultrasonography revealed post void residue of 80 ml. The patient did not have a history of previous trauma, catheterization, prior instrumentation, or any infective urethritis. A urine cytology was negative. A biopsy from stricture was suggestive of focal edema, myxoid changes, focal mild infiltrates of plasma cells, lymphocytes and eosinophils, and fibrosis without any atypical cells. An optical internal urethrotomy was done in April 2008. Foley's catheter was removed after 10 days. Presently, the patient is able to void freely with an adequate stream of urine.

## DISCUSSION

Bleomycin sulphate is composed of a mixture of cytotoxic glycopeptide antineoplastic antibiotic.[3] The cytotoxic effects result from the formation of oxygen free radicals which then cause single- and double-strand DNA breaks.

**Figure 2:**
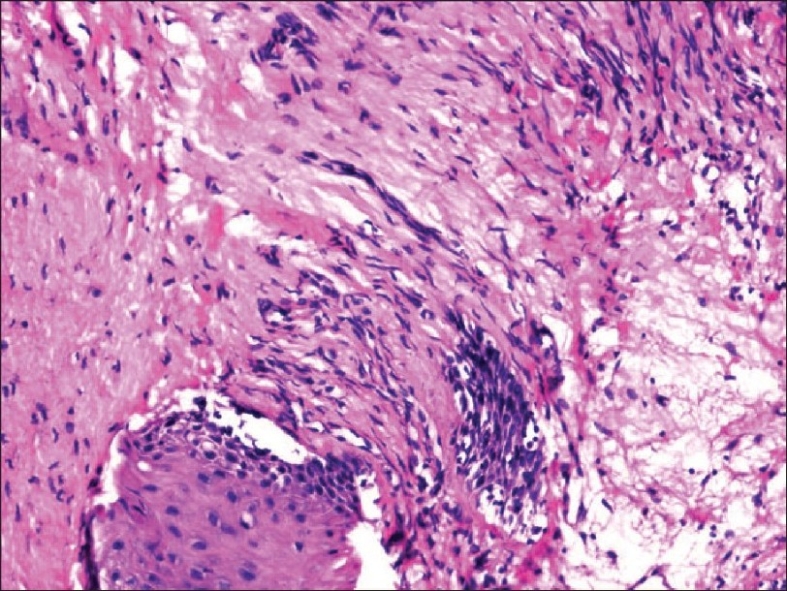
Urethral stricture-fibrosis with inflammatory cell infiltration

**Figure 3:**
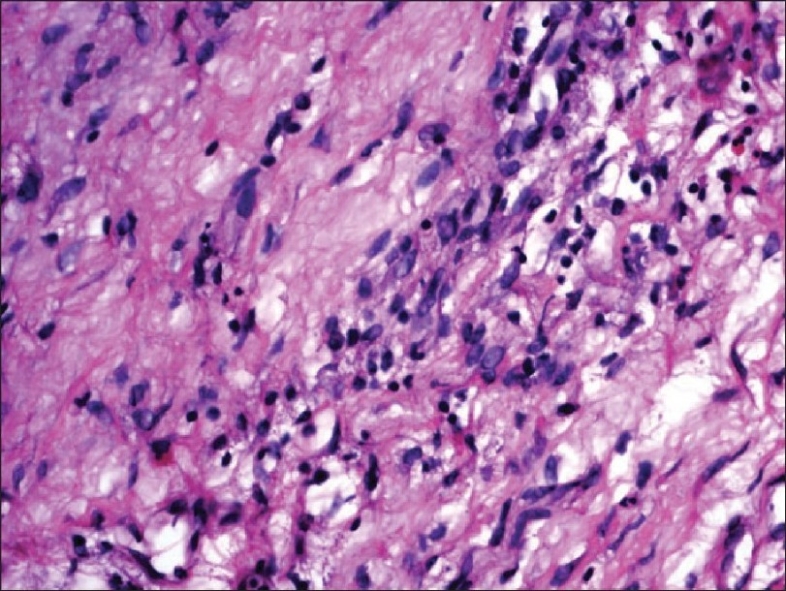
Urethral stricture-dense inflammatory cell infiltration

Elimination of bleomycin is primarily via the kidneys and approximately 60% to 70% of an administered dose is excreted unchanged through the urine. The dose-limiting toxicity of bleomycin is the induction of pulmonary toxicity leading to fibrosis. The central event is endothelial cell damage to the lung vasculature that results from the induction of various cytokines and the formation of oxygen free radicals. The most important cytokine involved in fibrosis is transforming growth factor beta (TGF beta).[4] Mucocutaneous toxicity presents as mucositis, erythema, hyperpigmentation, induration, hyperkeratosis, and skin peeling that may progress to ulceration. This usually develops in the second or third week of treatment and after a cumulative dose of 150 to 200 units of drug.[5] Levels of bleomycin hydrolase are low in the lung and skin tissue, perhaps offering an explanation as to why these normal tissues are more commonly affected by bleomycin.[6] Our patient developed dysuria without any evidence of infection after two cycles of chemotherapy. In our case, the patient received a total of 210 units of bleomycin. On receiving this dose, he developed pulmonary toxicity. The possibility of increased susceptibility to bleomycin toxicity may be considered. But he did not have any risk factors such as elderly age, smoking, impaired renal function, radiotherapy, or increased drug dose.[7] The patient had burning micturition after the second cycle of chemotherapy and the urine was sterile at that time. He did not have any difficulty in passing urine. This may be due to drug induced mucositis (urethritis). The subsequent healing might have resulted in stricture formation. After seven cycles, he developed difficulty in passing urine and had a poor stream of urine. He did not have any infective urethritis, including sexually transmitted diseases such as gonorrhoea, prior instrumentation or catheterisation, or trauma. It is very unlikely for a congenital stricture to present in this fashion and at this age.[8] Histology of the stricture also provides evidence towards an inflammatory reaction with edema and fibrosis [Figures 2 and 3]. In the pathogenesis of bleomycin-induced pulmonary fibrosis, inflammatory cells and other immune cells like neutrophils infiltrate and subsequent to the release of cytokines, fibrosis ensues.[9] Such a histologic picture is not seen in congenital stricture. It is unlikely to have any other cause in a sexually inactive individual. Moreover, a significant amount of bleomycin is excreted through the urine. Intratracheal instillation of bleomycin can induce pulmonary fibrosis in experimental mice.[10] This corroborative evidence points toward bleomycin-induced mucositis of the urethra as the probable etiological factor in our patient.

Probably, many similar conditions may remain asymptomatic in some patients receiving bleomycin. Other than the circumstantial evidence and follow-up study of all patients receiving bleomycin, it is almost impossible to prove objectively. Probably, urethral instillation studies in experimental animals may be needed to prove or disprove this theory. There is no similar case reported in English literature.

## CONCLUSION

Bleomycin-induced pulmonary fibrosis is a well-known side effect of bleomycin. Though mucocutaneous reactions occur with bleomycin, urethritis and urethral stricture have not been reported so far. In this case, the evidence suggests the possibility of bleomycin-induced urethral stricture.
